# Immediate Effects of Medially Posted Insoles on Lower Limb Joint Contact Forces in Adult Acquired Flatfoot: A Pilot Study

**DOI:** 10.3390/ijerph17072226

**Published:** 2020-03-26

**Authors:** Yinghu Peng, Duo Wai-Chi Wong, Yan Wang, Tony Lin-Wei Chen, Qitao Tan, Zhenxian Chen, Zhongmin Jin, Ming Zhang

**Affiliations:** 1 Department of Biomedical Engineering, Faculty of Engineering, The Hong Kong Polytechnic University, Hong Kong 999077, China; 18041923r@connect.polyu.hk (Y.P.); duo.wong@polyu.edu.hk (D.W.-C.W.); annie.wang@connect.polyu.hk (Y.W.); tony.l.chen@connect.polyu.hk (T.L.-W.C.); matthew.tan@connect.polyu.hk (Q.T.); 2 The Hong Kong Polytechnic University Shenzhen Research Institute, Shenzhen 518057, China; 3 Key Laboratory of Road Construction Technology and Equipment (Ministry of Education), School of Mechanical Engineering, Chang’an University, Middle-section of Nan’er Huan Road, Xi’an 710064, China; zhenxian_chen@yeah.net; 4 State Key Laboratory for Manufacturing System Engineering, School of Mechanical Engineering, Xi’an Jiaotong University, No.28, Xianning West Road, Xi’an 710049, China; zmjin@xjtu.edu.cn

**Keywords:** flatfoot, insole, pes planus, gait analysis, multibody simulation

## Abstract

Flatfoot is linked to secondary lower limb joint problems, such as patellofemoral pain. This study aimed to investigate the influence of medial posting insoles on the joint mechanics of the lower extremity in adults with flatfoot. Gait analysis was performed on fifteen young adults with flatfoot under two conditions: walking with shoes and foot orthoses (WSFO), and walking with shoes (WS) in random order. The data collected by a vicon system were used to drive the musculoskeletal model to estimate the hip, patellofemoral, ankle, medial and lateral tibiofemoral joint contact forces. The joint contact forces in WSFO and WS conditions were compared. Compared to the WS group, the second peak patellofemoral contact force (*p* < 0.05) and the peak ankle contact force (*p* < 0.05) were significantly lower in the WSFO group by 10.2% and 6.8%, respectively. The foot orthosis significantly reduced the peak ankle eversion angle (*p* < 0.05) and ankle eversion moment (*p* < 0.05); however, the peak knee adduction moment increased (*p* < 0.05). The reduction in the patellofemoral joint force and ankle contact force could potentially inhibit flatfoot-induced lower limb joint problems, despite a greater knee adduction moment.

## 1. Introduction

Nearly one-quarter of the adult population suffers from flexible flatfoot (or pes planus) [1,2,3]. Flexible flatfoot is characterized by the collapse of the medial longitudinal arch upon weightbearing and is often accompanied by excessive rearfoot eversion and forefoot abduction [1,4]. Foot posture is normally supported by the foot muscles and plantar fascia [5]. When the medial longitudinal arch is flattened and rearfoot eversion increases in flatfoot subjects, tensional force increases in the plantar fascia [6]. Patients with flatfoot also show abnormal alteration in foot muscle morphology [7]. Those reasons could cause flatfoot to progress into ankle pain and plantar fasciitis [8,9].

Flatfoot would induce other proximal joint problems, such as patellofemoral pain, owing to the coupled excessive foot pronation [8,10]. Razeghi et al. [11] reported that excessive pronation in the subtalar joint produces a large patellofemoral force. In addition, excessive eversion of the hindfoot excessively rotates the tibia. This could change the direction of the patellofemoral force and reduce the contact area of the patellofemoral joint, thereby applying extra stress to the joint during weightbearing [10,12]. Previous clinical studies also showed that flatfoot posture is associated with a greater risk of knee and lower extremity pain [3,8].

Foot orthoses can improve stability and facilitate pain relief by modifying lower limb alignment [13,14,15,16,17,18]. A recent review conducted by Desmyttere, Hajizadeh, Bleau and Begon [18] examined the influence of the orthosis design on the kinematics of the lower extremity. They discovered that medial posting insoles were the most effective foot orthoses to reduce the rearfoot eversion angle for adults with flatfoot. However, this did not come into a consensus for joint moments. Telfer, Abbott, Steultjens and Woodburn [16] found that increasing the medial posting wedge elevated the knee adduction moment while Kosonen, Kulmala, Müller and Avela [14] did not find a significant difference in the knee adduction moment during walking. Hsu et al. [19] adopted a medial posting insole in the forefoot and rearfoot and found that the foot orthosis significantly increased the knee internal abduction moment and reduced the ankle inversion moment and hip abduction moment. Regardless, previous studies have evaluated the biomechanics of foot orthoses for flatfoot by joint angles or moments [14,15,16,18,19], whereas the joint contact force represents a more direct parameter used to reveal the mechanism of secondary knee joint problems, such as pain and cartilage wear [20]. However, measurement in vivo was difficult owing to the invasiveness. 

The musculoskeletal (MSK) model or musculoskeletal multi-body simulation has been used to estimate the lower limb joint contact forces [21,22]. Previous studies have adopted the model to investigate the joint contact forces for the hip [23], patellofemoral [24], tibiofemoral [25] and ankle joints [22] in daily activities. This tool also enabled a relatively accurate prediction of the medial and lateral tibiofemoral joint forces, which could be used to investigate the force changes in patients with medial knee osteoarthritis [26,27]. Although much work has been done on the lower limb joint contact forces in various conditions, we found that no studies have investigated the effects of foot orthosis on the lower limb joint contact forces for the adult with flatfoot through musculoskeletal multibody model after a review of the related literature. Thus, using the MSK model, we aim to evaluate the influence of the foot orthosis, particularly the arch support insole with medial forefoot posting, on the estimated joint contact forces of the lower limb for adults with flatfoot during level walking. We hypothesized that the foot orthosis could significantly reduce the hip, ankle, patellofemoral, and medial and lateral tibiofemoral joint contact forces.

## 2. Materials and Methods

### 2.1. Participants

A priori power analysis software (G*power 3.0.10, Universität Düsseldorf, Germany) was used to calculate the minimum sample size [28]. For the parameters before and after foot orthosis intervention on the same sample, a statistical power of 0.8 and an alpha level of 0.05 was chosen, respectively. To detect changes in matched pairs with unknown effect sizes, this study adopted a medium effect size of 0.8. With the chosen parameters, a sample size of at least 15 subjects was required using power analysis software. Fifteen young adults (nine men and six women) with flatfoot were recruited from Hong Kong Polytechnic University from 2018 October to 2020 March. Previous studies adopted many methods to categorize the foot posture [29]. In this study, an ink-footprint method [30] was used as it is reliable, cheap, easy, and requires almost no expertise. The foot posture of each participant was evaluated by measuring the Harris mat footprint during half weightbearing [31]. The inclusion criteria were (1) an arch index of ≥ 0.28 for both feet [30], (2) 18 to 25 years old, and (3) not overweight (BMI  >  30 kg·m^−2^). The exclusion criteria included (1) neuromuscular disease, (2) biomechanical abnormalities other than flatfoot and complications affecting walking ability and performance, and (3) foot orthoses use or physiotherapy in the last 6 months. To facilitate footwear fitting, the foot size of each participant was measured using the Brannock device before the experiments. Ethical approval was approved by the Human Subjects Ethics Sub-Committee of the Hong Kong Polytechnic University (Number: HSEARS20150121003). The consent form and information form for data collection and research contents were provided and signed prior to the experiment.

### 2.2. Equipment and Procedure

**Figure 1:**
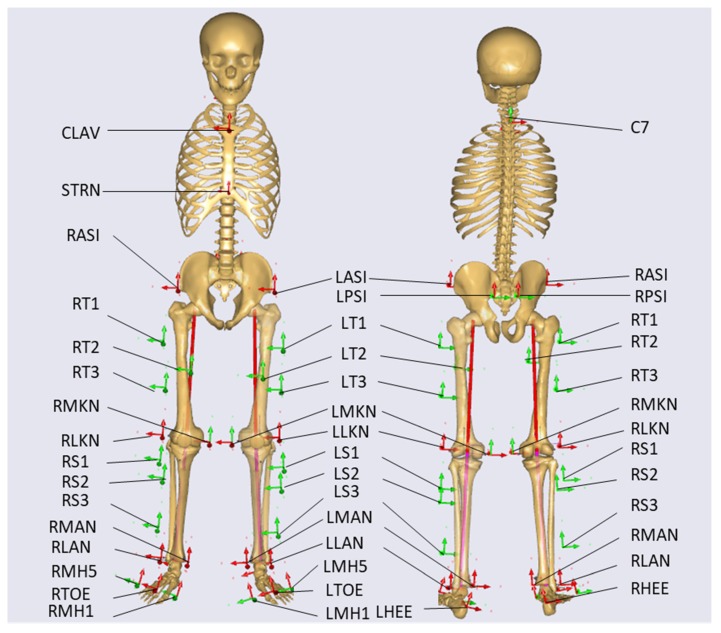
Locations and names of the reflective markers.

A three-dimensional motion capture system, including eight cameras (Vicon, Oxford Metrics Ltd., Oxford, England) and four force plates (OR6, AMTI, Watertown, United States), was used to capture marker trajectories and ground reaction forces. The data were collected synchronously with a sampling frequency of 100 Hz and 1000 Hz, respectively. A modified plug-in gait model [32] was used to capture the gait posture (Figure 1). The gait model uses the reflective markers placed on the anatomical locations and participants’ parameters to calculate the joint angles. A total of thirty-five markers were attached to landmark locations on the subject, including the xiphoid process of the sternum, jugular notch, 7th cervical vertebrae, anterior/posterior superior iliac crest, three skin-mounted markers orientated in a triangle placed of the middle thigh/shank, lateral/medial femoral epicondyles, lateral /medial malleoli, calcaneal tuberosity and the head of the 1st, 2nd and 5th metatarsals. Compared to the plug-in gait model [32], additional markers on the medial femoral epicondyles, medial malleoli and the head of the 1st and 5th metatarsals could be used to estimate the static joint locations easily and to scale the bone length during scaling process.

**Figure 2:**
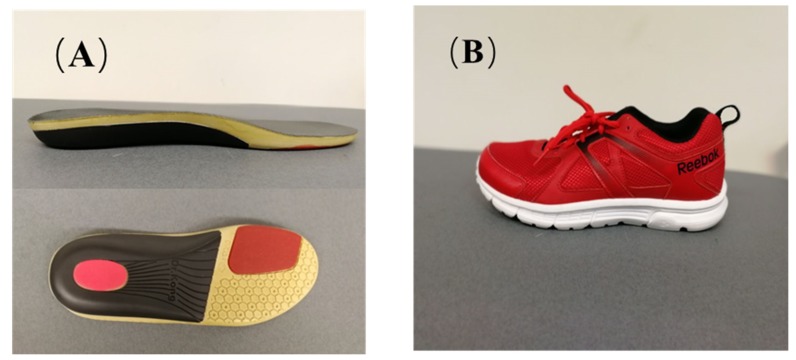
The view of foot insole (**A**) and shoe (**B**).

Gait analysis was performed randomly on all participants in two conditions: walking with shoes and foot orthoses (WSFO) and walking with shoes (WS). They were equipped with the same type/brand of running shoes (Reebok Run Supreme 4.0, Reebok, Boston, United States) and foot orthoses (Universal Flat Foot, Dr Kong, HK, China), as shown in Figure 2. According to the previous study [18], medial forefoot or both medial forefoot and rearfoot posting insoles could effectively reduce the rearfoot eversion angle for adults with flatfoot, so foot orthosis with arch support and forefoot posting insoles were used in this study. The foot orthoses were prefabricated insoles with a 3-cm thick medial arch support and 6° inclined medial forefoot posting. The insoles were made of fabric with embedded cushioning silicon at the heel region.

Prior to the experiment, the participants were given a few minutes to become comfortable with the footwear and the environment. During the gait experiment, the participants were asked to walk on a 10-m pathway at a self-selected comfortable speed. Prior to level walking trials, static data of the marker trajectories were collected for each participant. For each subject, walking trials on the right foot were collected. During the walking trials, WSFO and WS conditions were performed in random order. Six successive walking trials were collected for each condition. The trials were considered successful when their footsteps were placed entirely within the force plates. For the walking trials, the force plate data and marker trajectory data were collected. The data collected was then output as C3D format, which could be used in the musculoskeletal multibody model.

### 2.3. Musculoskeletal Model

A commercial software package of the MSK model, Anybody (AnyBody Technology, Aalborg, Denmark, version 6.0.5), was used along with a built-in generic lower limb MSK model [21]. The generic MSK model is based on the anthropometric database of Twenty Lower Extremity Model version 1.1, including the spherical hip, hinged patellofemoral, tibiofemoral, ankle, and subtalar joints and approximately 160 muscle units [33]. The MSK was driven by the experimental data of the gait analysis.

**Figure 3:**
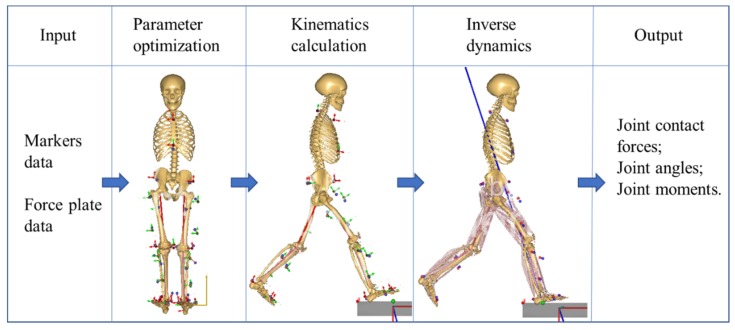
The workflow of the musculoskeletal multibody model.

Three steps were performed to estimate the joint contact force in this study according to the previous study [34]. Marker trajectories and force plate data were used to scale the model and calculate the kinematics and kinetics of the lower limb joint. The force plate data were filtered with a cut-off frequency of 100 Hz. Marker trajectories were filtered with a cut-off frequency of 7 Hz via a fourth-order Butterworth low-pass filter. More details about the MSK multibody modeling are shown in Figure 3. First, the patient-specific MSK model was obtained by scaling the generic model with the static marker positions during the parameter optimization [34]. In this step, the ratio of the experimental markers and model markers could be calculated to scale the bone length. Muscle insertion points and muscle moment arms were scaled to match each subject’s segment lengths. The length-mass-fat scaling criterion [21] was assigned to the simulation system, thus distributing the body mass to the related part of the body. Second, dynamic walking trials were used as inputs to drive the model. In this step, the position, velocities, and acceleration of the segments, as well as the joint angles of the lower limb, could be calculated. Finally, the calculated joint angles, velocities and accelerations in the inverse kinematics and force plates data were used as inputs to drive the musculoskeletal model during the inverse dynamics step. In this step, the tibiofemoral joint was modeled as a spherical joint to allow rotations. To account for abduction/adduction and internal/external resistance provided by the knee ligament structure, two external moments in both directions were included. The muscles passing through the knee only balance the flexion/extension. An equilibrium problem in the frontal between the total tibiofemoral contact force and the adduction/abduction moment was solved to estimate the medial and lateral tibiofemoral contact forces [35]. In this step, hip, patellofemoral and ankle joint contact forces could be calculated simultaneously. The joint contact forces were estimated by the third-order polynomial muscle recruitment criteria, which minimize a cost function G, subject to the dynamic equilibrium equations [35,36].

(1) $$\underset{f}{\min}G\left(f^{\left(M\right)}\right)={\sum_{i=1}^{n^{\left(M\right)}}n_{i}\left(\frac{f_{i}^{\left(M\right)}}{s_{i}}\right)}^{3}$$

(2) $$\begin{array}{l} Cf=r\\ 0\leq f_{i}^{\left(M\right)}\leq s_{i},\;i=1\ldots n^{\left(M\right)} \end{array}$$

Here, $f_{i}^{\left(M\right)}$ represents the ith muscle force; $s_{i}$ is the isometric strength of the ith muscle; $n_{i}$ is the normalization factor, which reflects the sub-divided muscle model; $n^{\left(M\right)}$ is the number of muscles; C is the coefficient matrix of equilibrium equations; f is a vector of the muscle and joint forces, and r contains all external loads and inertia forces.

Each muscle-tendon unit was assigned an isometric muscle strength which was calculated by multiplying the physiological cross-sectional area (PCSA) by a factor of 27 N/cm^2^ [35,36]. The PCSA was obtained from cadaver-based studies [21,22]. Additionally, to ensure that muscles only pulled and did not push, non-negativity constraints were used for the muscles.

### 2.4. Statistical Analysis

The statistical analysis was performed using SPSS (Version 22.0, IBM, Chicago, IL, USA). The normality of the data distributions was verified using the Shapiro-Wilk test (*p* > 0.05). Paired t-tests were performed to compare the walking velocities, peak joint angles and moments, joint contact forces and vertical ground reaction forces between the WS and WSFO conditions. The calculated joint moments and forces were normalized to the bodyweight times the body height (BW·BH) and the bodyweight (BW), respectively. The effect size (ES) was indicated by the standardized mean differences (SMD) and was categorized as trivial, small, medium and large for 0 ≤ ES ≤ 0.2, 0.2 ≤ ES ≤ 0.5, 0.5 ≤ ES ≤ 0.8, and ES ≥ 0.8 [37]. The significance level was set at 0.05.

## 3. Results

The average age of the participants was 21.7 ± 1.2 years. The average height and weight were 168.5 ± 7.3 cm and 57.8 ± 7.9 kg, respectively. There was no significant difference in the walking speed between WS and WSFO (*p* > 0.05). There was also no significant difference in the two peaks of the vertical ground reaction forces between WS and WSFO (*p* > 0.05).

**Figure 4:**
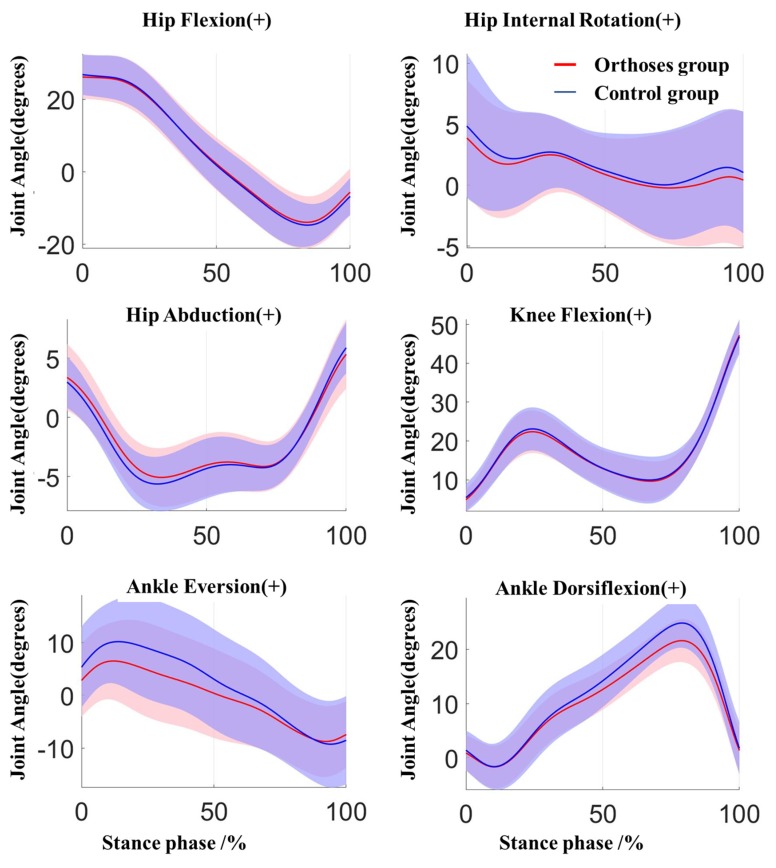
Joint kinematics of the lower limb in the orthoses group (red) and control group (blue) during stance phase. Shaded area±1 std.

For the joint angles, there were significant main effects for ankle dorsiflexion and eversion. A significant reduction in the ankle dorsiflexion (*p* = 0.002, ES = 1.015) and ankle eversion (*p* = 0.001, ES = 1.468) was presented in the WSFO condition when compared to WS condition. More details about the peak joint angles between the WSFO and WS group are listed in Table 1. The joint angles of the lower limb during the stance phase are shown in Figure 4.

**Figure 5:**
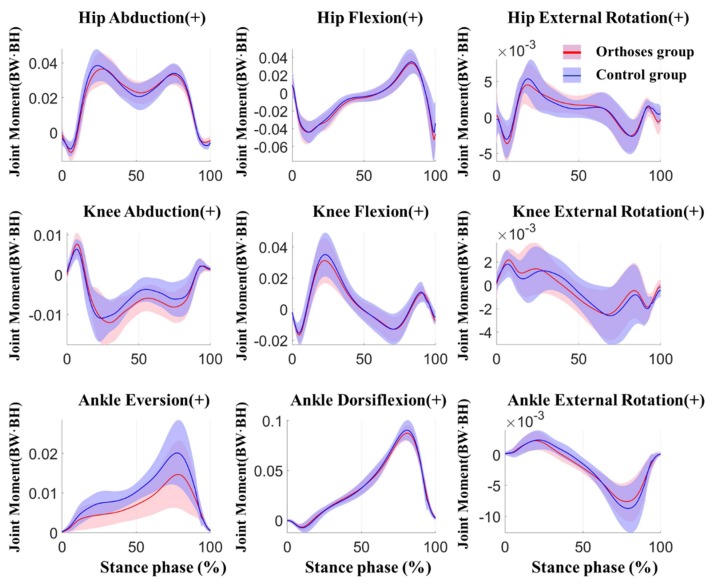
Joint moments of the lower limb in the orthoses group (red) and control group (blue) during stance phase. Shaded area±1 std.

For the joint moments, there were significant main effects for the knee and ankle joint. A significant increase in the first peak knee adduction moment (*p* = 0.007, ES = 0.822) and second peak knee adduction moment (*p* = 0.016, ES = 0.707) was present in the WSFO condition when compared to WS condition. As for the ankle joint, a significantly lower peak ankle eversion moment (*p* = 0.002, ES = 1.627) and lower peak ankle internal moment (*p* = 0.014, ES = 0.728) was present in the WSFO condition. More details about the peak joint moments in three planes are listed in Table 2. The joint moments of the hip, knee, and ankle versus time during the stance phase between WSFO and WS (control) are compared in Figure 5.

**Figure 6:**
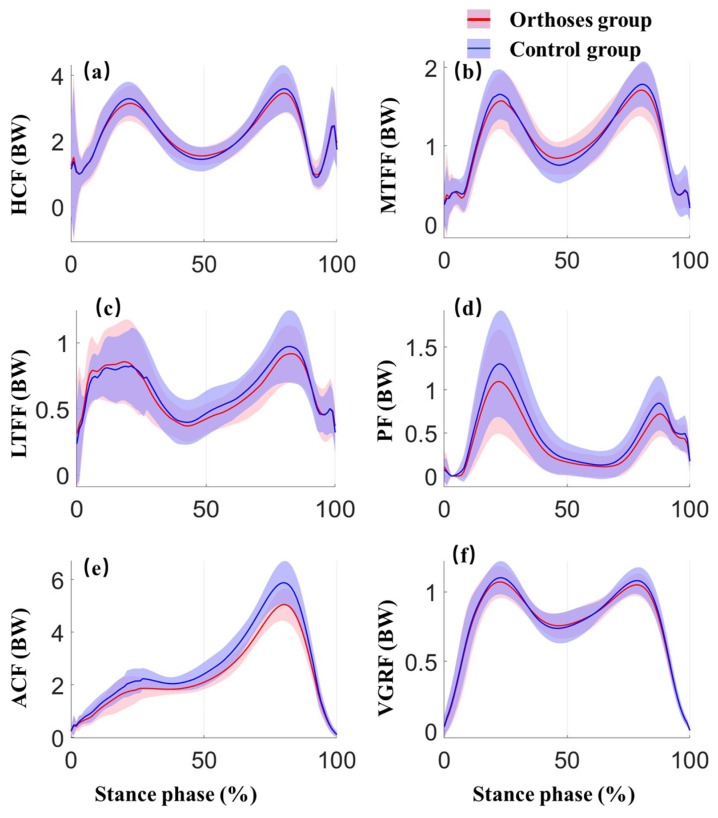
Joint contact forces of the lower limb and ground reaction forces in the orthoses group (red) and control group (blue) during stance phase. Shaded area±1 std (**a**) HCF: hip contact force (**b**) MTCF: medial tibiofemoral contact force (**c**) LTCF: lateral tibiofemoral contact force (d)PCF: patellofemoral contact force (**e**) ACF: ankle contact force (**f**) VGRF: Vertical ground reaction force.

As for the lower limb joint contact forces, there were significant effects for ankle joint and patellofemoral joint. The participants experienced lower second peak patellofemoral contact force (*p* < 0.05) as well as lower peak ankle contact forces (*p* < 0.05) with the foot orthoses compared to the WS conditions. Significant decreases were observed in the second peak of patellofemoral contact forces by 10.2% (*p* = 0.022, ES = 0.663) and peak ankle contact forces by 6.8% (*p* = 0.004, ES = 1.03) in WSFO condition when compared to WS condition. More details about the peak contact forces during the stance phase are listed in Table 3. The lower limb joint contact forces and ground reaction forces versus time during the stance phase between WSFO and WS (control) are compared in Figure 6.

## 4. Discussion

In this study, we utilized the MSK model approach to investigate the influence of the foot orthosis (medial arch support with forefoot medial posting) on the biomechanics of the lower extremity for adults with flatfoot. Compared to the traditional joint angle or moment parameters, the estimated joint force could provide a relatively direct interpretation of clinical manifestation, which is difficult to measure in vivo [38,39]. Thus, the medial and lateral contact force of the tibiofemoral and the patellofemoral joints were estimated. Our study suggested that the orthosis significantly reduced the peak ankle contact force (*p* = 0.004, ES = 1.03) and second peak of the patellofemoral contact force (*p* = 0.022, ES = 0.663). However, there was no significant difference for the peak hip joint contact forces (*p* > 0.05) as well as the medial (p > 0.05) and lateral (p > 0.05) tibiofemoral joint contact force in the WSFO and WS groups.

In this study, the foot orthosis significantly reduced the peak total ankle contact forces (*p* = 0.004, ES = 1.03), ankle eversion moment (*p* = 0.002, ES = 1.627), and ankle internal rotational moment (*p* = 0.014, ES = 0.728) when compared to the WS condition. The tibialis posterior is an important stabilizer and plays a major role in supporting the medial longitudinal arch and inversion [40]. Failure or insufficiency of the tibialis posterior could be a cause of flatfoot [41]. The orthosis with arch support could sustain the medial longitudinal arch and control excessive pronation. The orthosis assists in some functions of the tibialis posterior, which could probably be failing among the flatfoot adults. The significant reduction in the peak ankle eversion and internal rotation moment facilitated by the orthosis could alleviate further pronation and progression [18], which could be difficult to obtain by surgical intervention alone [42]. Moreover, the orthosis decreased the functional demand of the tibialis posterior, and this could have caused a significant reduction of the ankle joint contact force, as supported by previous literature [43]. Furthermore, excessive rearfoot eversion could also impose additional load on the tibialis posterior muscle [44,45].

Our finding suggested that foot orthosis significantly reduced the second peak of the patellofemoral joint force (*p* = 0.022, ES = 0.663). Flatfoot patients are more vulnerable to develop patellofemoral pain syndromes, which are believed to be associated with the coupling of abnormal foot alignment and kinematics resulting in additional stress to the knee [8]. Individuals with pronated foot have a larger peak patellofemoral contact force [11]. The foot orthosis reduced the patellofemoral joint force, which could potentially inhibit flatfoot-induced knee joint problems. Although the knee adduction moment was significantly increased (*p* < 0.05) by the foot orthosis, there was no significant difference between WSFO and WS conditions for the medial and lateral tibiofemoral contact forces (*p* > 0.05). The knee adduction moment was one of the most common parameters used to evaluate knee kinetics; however, a large joint moment does not imply a large contact force [46,47]. The information on the medial and lateral compartmental joint forces provides useful supplementary information to evaluate interventions for common knee pathologies, especially knee osteoarthritis [20].

A number of studies have examined foot kinematics and kinetics after orthosis use [14,15,16,18,48]. Regarding the joint angles, the foot orthosis significantly reduced the peak hindfoot eversion by 3.8° (*p* = 0.001, ES = 1.468), which was consistent with previous studies using a medial forefoot or medial full-length insole [49,50]. In addition, there was no significant difference in the vertical ground reaction force and walking speed, which was in line with Leung study [51]. However, existing research presented mixed results on joint moments [14,52] which could be ascribed to the differences in the orthotic design, such as the stiffness and arch support height [13].

Several limitations should be discussed. This study focused on the immediate effect of the foot orthosis, and the prediction outcome may be influenced by the body build, gender, knee alignment, and symptoms. The joint contact force prediction was based on a validated MSK platform [21]. We did not conduct a specific set of validations for our samples. The MSK model assumed a scaled geometry of the skeleton with rigid bones and the encapsulated tissue was not considered. A three-dimensional anatomically detailed finite element model could provide further insights by considering deformation and interactions among the soft tissue [53], as well as facilitating a versatile platform for the parametric analysis of the footwear and foot disorders in the elderly with flatfoot [54,55,56]. Finally, the relatively small sample size was another limitation in this study. The relatively small sample size could produce insufficient statistical power and may hinder the generalizability of the findings. The implications of this pilot study shall be noted cautiously. Additional, work is required to further understand the biomechanical effects and clinical effects of different insole designs on asymptomatic and symptomatic flatfoot subjects of different ages, especially for the elderly.

## 5. Conclusions

In this study, we investigated the influence of foot orthoses (medial arch support with medial forefoot posting) on the biomechanics of the lower extremity for adults with flatfoot. The peak ankle joint contact force and second peak patellofemoral joint contact force were significantly lower in the WSFO group. Moreover, the peak ankle eversion angle and ankle eversion moment were also reduced by the foot orthosis. The reduction in the patellofemoral joint force and ankle contact force could potentially inhibit flatfoot-induced lower limb joint problems.

## Figures and Tables

**Table 1:** Peak joint angles between two walking conditions during stance (°).

Parameter	WSFO Mean (SD)	WS Mean (SD)	*p*−Value	ES (95% CI)
Hip flexion	26.827 (6.373)	27.256 (5.948)	0.443	0.204 (−0.736,1.593)
Hip extension	−14.222 (7.347)	−15.025 (5.884)	0.257	0.305 (−2.263,0.656)
Hip abduction	5.868 (2.796)	6.095 (2.318)	0.421	0.214 (−0.361,0.815)
Hip adduction	−5.563 (2.449)	−5.957 (2.355)	0.072	0.502 (−0.828,0.041)
Hip external rotation	5.181 (4.157)	6.225 (4.622)	0.145	0.398 (−0.407,2.494)
Hip internal rotation	−2.212 (4.909)	−2.309 (4.547)	0.871	0.043 (−1.361,1.167)
Knee flexion	47.687 (4.112)	46.82 (4.539)	0.223	0.329 (−2.326,0.592)
Ankle dorsiflexion	22.848 (4.163)	24.734 (4.576)	0.002 ^1^	1.015 (0.857,2.915)
Ankle plantarflexion	−2.514 (3.917)	−2.663 (4.612)	0.81	0.063 (−1.454,1.155)
Ankle eversion	7.06 (6.675)	10.886 (6.764)	0.001 ^1^	1.468 (2.382,5.27)
Ankle inversion	−9.521 (6.931)	−9.734 (8.737)	0.85	0.05 (−2.588,2.161)

WSFO: Walking with shoes and orthoses; WS: Walking with shoes; ES: Effect size. ^1^ Significant difference compared to WS (walking with shoes) at *p* < 0.05

**Table 2:** Peak amplitude of joint moments between two walking conditions during stance (bodyweight times the body height, BW·BH).

Parameter		WSFO Mean (SD)	WS Mean (SD)	*p*−Value	ES (95% CI)
Hip	First Abduction	−0.039 (0.01)	−0.041 (0.009)	0.106	0.446 (−0.003,0)
	Second Abduction	−0.034 (0.006)	−0.034 (0.006)	0.193	0.353 (−0.001,0)
	External Rotation	0.006 (0.003)	0.005 (0.002)	0.362	0.243 (−0.002,0.001)
	Internal Rotation	−0.006 (0.002)	−0.006 (0.002)	0.357	0.246 (−0.001,0)
	Flexion Rotation	0.061 (0.016)	0.058 (0.017)	0.443	0.204 (−0.013,0.006)
	Extension Rotation	−0.037 (0.007)	−0.039 (0.01)	0.191	0.355 (−0.005,0.001)
Knee	First Adduction	−0.015 (0.004)	−0.013 (0.004)	0.007 ^1^	0.822 (0,0.003)
	Second Adduction	−0.009 (0.004)	−0.008 (0.003)	0.016 ^1^	0.707 (0,0.002)
	External Rotation	0.003 (0.002)	0.003 (0.001)	0.592	0.142 (0,0)
	Internal Rotation	−0.003 (0.002)	−0.003 (0.002)	0.224	0.329 (0,0)
	Flexion	0.019 (0.006)	0.019 (0.007)	0.932	0.022 (−0.002,0.002)
	Extension	−0.032 (0.015)	−0.036 (0.015)	0.18	0.364 (−0.009,0.002)
Ankle	Eversion	0.015 (0.008)	0.021 (0.008)	0.002 ^1^	1.627 (0.003,0.007)
	Inversion	0.001 (0.001)	0.002 (0.001)	0.101	0.453 (0,0.001)
	External	0.002 (0.001)	0.003 (0.001)	0.083	0.482 (0,0)
	Internal	−0.008 (0.003)	−0.009 (0.004)	0.014 ^1^	0.728 (−0.002,0)
	Dorsiflexion	0.089 (0.008)	0.091 (0.01)	0.08	0.487 (0,0.005)
	Plantarflexion	−0.008 (0.004)	−0.009 (0.005)	0.182	0.363 (−0.002,0.001)

WSFO: Walking with shoes and orthoses; WS: Walking with shoes; ES: Effect size. ^1^ Significant difference compared to WS (walking with shoes) at *p* < 0.05

**Table 3:** Peak amplitude of joint force and vertical ground reaction force between two walking conditions during stance (BW).

Parameter		WSFO Mean (SD)	WS Mean (SD)	*p*−Value	ES (95% CI)
Hip	First peak	3.286 (0.502)	3.355 (0.523)	0.42	0.215 (−0.109,0.248)
	Second peak	3.562 (0.527)	3.666 (0.675)	0.201	0.347 (−0.062,0.269)
Tibiofemoral joint	First medial peak	1.617 (0.324)	1.684 (0.356)	0.249	0.31 (−0.052,0.185)
	Second medial peak	1.737 (0.318)	1.81 (0.313)	0.262	0.302 (−0.061,0.208)
	First lateral peak	0.894 (0.292)	0.927 (0.264)	0.503	0.177 (−0.07,0.136)
	Second lateral peak	0.904 (0.224)	0.994 (0.291)	0.095	0.462 (−0.018,0.199)
Patellofemoral joint	First peak	1.179 (0.63)	1.324 (0.686)	0.153	0.391 (−0.061,0.352)
	Second peak	0.78 (0.259)	0.869 (0.334)	0.022 ^1^	0.663 (0.015,0.164)
Ankle	Peak	5.521 (0.627)	5.925 (0.882)	0.004 ^1^	1.03 (0.187,0.621)
Ground force	First peak	1.099 (0.127)	1.113 (0.128)	0.458	0.197 (−0.025,0.053)
	Second peak	1.066 (0.081)	1.074 (0.08)	0.238	0.318 (−0.006,0.023)

WSFO: Walking with shoes and orthoses; WS: Walking with shoes.ES: Effect size. ^1^ Significant difference compared to WS (walking with shoes) at *p* < 0.05.
